# Management of Advanced Heart Failure in the Elderly: Ethics, Economics, and Resource Allocation in the Technological Era

**DOI:** 10.1155/2012/524961

**Published:** 2012-12-05

**Authors:** Keith M. Swetz, John M. Stulak, Shannon M. Dunlay, Ellin F. Gafford

**Affiliations:** ^1^Division of General Internal Medicine, Department of Medicine, Section of Palliative Medicine, Mayo Clinic, 200 First Street SW, Rochester, MN 55905, USA; ^2^Division of Cardiovascular Surgery, Mayo Clinic, 200 First Street SW, Rochester, MN 55905, USA; ^3^Division of Cardiovascular Medicine, Department of Medicine, Mayo Clinic, 200 First Street SW, Rochester, MN 55905, USA; ^4^Palliative Medicine, Ross Heart Hospital, Wexner Medical Center, The Ohio State University, Columbus, OH 43210, USA

## Abstract

Significant strides have been made in the durability, portability, and safety of mechanical circulatory support devices (MCS). Although transplant is considered the standard treatment for advanced heart failure, limits in organ availability leave a much larger pool of recipients in need versus donors. MCS is used as bridge to transplantation and as destination therapy (DT) for patients who will have MCS as their final invasive therapy with transplant not being an option. Despite improvements in quality of life (QOL) and survival, defining the optimal candidate for DT may raise questions regarding the economics of this approach as well as ethical concerns regarding just distribution of goods and services. This paper highlights some of the key ethical issues related to justice and the costs of life-prolonging therapies with respect to resource allocations. Available literature, current debates, and future directions are discussed herein.

## 1. Identifying the Problems in a Complex Landscape

Heart failure is a serious and costly health care issue globally. The prevalence of heart failure in the US is 5.7 million patients, with 670,000 new cases diagnosed annually and greater than 56,000 deaths attributable to it. Ten percent of patients have “advanced heart disease” costing an estimated 82.2 billion US dollars (USD) annually [1]. Previously heart transplantation was the only definitive treatment option for end-stage heart failure, but a limited donor pool has led to development of alternative strategies. Mechanical circulatory support (MCS) had been utilized increasingly over the last 20 years as *destination therapy* (DT), in which patients receive MCS, but are not considered transplantation candidates. While the costs of DT have improved [2], as have survival and quality of life (QOL) [3], questions remain regarding how broadly DT should be utilized in the elderly, and there is a need for guidance regarding age and appropriate patient selection [4]. There remain great opportunities and challenges to caring for an ever-aging global population. We believe these points warrant emphasis and further consideration beyond cost effectiveness or survival analyses alone.

 Improved survival has been a major impetus for MCS with initial studies demonstrating pulsatile flow devices achieved 52% 1-year survival compared to 25% with optimal medical management [5]. More recently, MCS design and durability have improved and 2nd-generation continuous-flow devices have more robust survival benefits than their predecessors [3, 6]. Work is ongoing to determine benefits of newer-generation centrifugal devices, as well as optimal timing of DT implant. Well-selected patients receiving DT can anticipate 75% two-year survival with improved QOL [7], which underscores the importance of appropriate patient selection in optimizing outcomes. While Medicare covers DT costs in the United States, other national health plans often only cover MCS for bridge to transplantation and do not even offer DT as a therapeutic option. In an era of rising healthcare costs, finite resources, and aging of the population, optimizing selection of patients for MCS and DT becomes increasingly important. 

## 2. Caring for the Sickest of the Sick

While data suggest candidates who are Interagency Registry for Mechanically Assisted Circulatory Support (INTERMACS) Profile 3 through 6 may benefit most from DT [7], an inherent pressure to offer DT to sicker (INTERMACS Profile 1-2) patients may be governed by what Norman Daniels has termed as *the rule of rescue*. Daniels postulates that if a patient is facing a life-and-death situation and there is a reasonable means of intervention to alter that course, then the goals of medicine tend to promote utilizing those means [8, 9]. This is challenging for the sickest patient as DT outcomes are inferior with worse INTERMACS profiles, or if a patient's medical condition cannot be optimized prior to DT [7, 10].

The US health care environment allows patients to be rescued (potentially at great financial cost), as this is often a societal expectation and systematic barriers are lacking to the contrary. Patient autonomy, in the context of an inherent perception of rescue as the norm may be a major driver, but may be at inherent tension with the justice. Optimally, principles need to be considered *prima facie*—considering each individual set of circumstances independently, unless a law or policy more clearly outlines steps to be taken. However, individualized medicine does not lend itself well to broad guideline establishment.

Identifying optimal timing of DT implantation is under investigation as some have suggested that worst outcomes result from waiting too long to receive DT (see REVIVE-IT and ROADMAP trials at http://www.clinicaltrials.gov/). This belief, however, may create a two-tiered system where the onus of serving the highest risk patients and offering *rescue* becomes a *de facto* obligation of tertiary/quaternary referral centers. Within US society, general moral priority is to help the sickest patients, as well as those for whom there are not alternatives other than comfort care only [9]. This is paradoxical as there are limits to what can be achieved by technology, and these limits must be recognized to make the system function optimally balancing a prevalent expectation of intervention, even when questionably beneficial.

## 3. A Public Debate Frames Some of the Challenges

A multidisciplinary panel recently presented a plenary session on some of the challenges to justice and fair allocation of MCS and differences between North American and European practices. Issues raised included optimal age of implantation, particularly with an ever-aging world population. While MCS has demonstrated value regarding survival, questions regarding QOL or general condition and functional status beyond hospital discharge may be more important endpoints. A recent study examined DT in an older cohort (age 70–79) compared to younger patients (age 60–69) [11]. The findings suggest that the older cohort can incur lower costs associated with DT, and that the older patients have similar mortality compared to the younger patients in their 60s (odds ratio 1.33 [0.74–2.41; *P* = 0.34]). However, while the older cohort appears to incur less cost, these patients may die sooner and thus utilize fewer resources. In this study, the older patients were discharged to nursing homes at higher rates and patient followup after discharge was not reported in detail. This raises an important question regarding whether patients who received DT were able to return to independent living—as this is often a major goal of DT in an older population. 

Others, such as Ross, have explored constructs such as “accountability for reasonableness” (A4R) in terms of considering use of scarce resources such as transplant [12]. A4R is a deliberative process to evaluate scarce resources and has been used to help determine optimal and ethical transplantation guidelines in Canada (unpublished analysis). While the consideration of A4R with transplantation was made within the context of a single-payer, socialized system (Canada) this nevertheless begs the question if such an approach is applicable beyond transplant and if A4R may be applied to DT. The A4R framework gathers key stake holders to review available data and make recommendations for best practice. Unfortunately in a pluralistic society, even the best recommendations or consensus statements may be challenging to implement universally, or even adopted from one nation or health care system to another. 

In the interim while we work towards an ideal allocation system for MCS, the data regarding the costs of MCS are worth considering. Upfront costs can be significant, as initial hospitalization for DT implantation is approximately 128,000 USD [13], improved from 210,000 USD with pulsatile-flow devices [14]. Despite dramatic reduction in the cost of post-DT care, as well as improved procedure-associated morbidity and mortality [2], costs are not inconsequential. While device cost is a fixed expense regardless of INTERMACS profile at implantation time, costs after implantation may widely vary, depending on major complications, or protracted hospitalization. This is why it is particularly important to select the optimal candidates for MCS, not just based on age, but on a host of physiologic and psychosocial factors that may be associated with good or suboptimal outcomes.

## 4. Do Cost-Effectiveness Analyses and QALYs Help Us Care for Patients Better?

 While cost analyses often try to quantify medical progress in terms of innovation, work has yet to be done to better couple financial and clinical outcomes. Recent DT cost-effective analyses utilized the concept of quality-adjusted life years (QALYs) [15], which attempt to couple a factor of function and quality of life with a factor of prolonged survival. While it is logical to incorporate these two important factors, they have been widely criticized by some regarding their ethical merit [16–19]. By definition, QALYs attempt to quantify the magnitude an intervention has on improving QOL or relative health over a given time period as a given cost. Health care decisions with low cost per QALY could be prioritized, while ones with higher cost per QALY might require additional scrutiny [9]. Unfortunately, such analyses aim to focus on the *process* associated with resource allocation rather than the *reasons* for those decisions. 

If the conditions for a just decision-making process are specified, it is conceivable that issues regarding individual application of such principles may be avoided. Some have suggested when decisions are made by a central arbitrating body (as in the United Kingdom), two major difficulties occur [9]. First, evidence may be lacking that spending money one way over another is beneficial (e.g., health promotion or technology), and second, even if cost-effectiveness evidence is strong, a given group may face ethical choices in determining optimal conditions in which to apply the therapy [9]. Focusing on the allocation processes versus individual cases may help to temper some concerns, but each individual case has context that may impact the likelihood of interventional benefit.

 Ethical concerns regarding the applicability of cost-effectiveness and the QALY include, (1) what defines the value of a life, (2) should the young have a different priority than the old, and (3) should the *rule of rescue* be noted when an obligation to save a life outweighs competing values [9, 16]. Six criteria have been proposed that should be met for QALYs to have ethical legitimacy in the clinical arena and within societal expectations [17]. These include, (1) quality of life can be accurately measured and used, (2) utilitarianism is acceptable, (3) equity and efficiency are compatible, (4) projections of community preferences can substitute for individual preferences, (5) the old have less “capacity to benefit” than the young, and (6) physicians will not use quality-adjusted life years as clinical maxims [17]. These points are well-articulated and have some degree of reasonableness, but are not universally accepted. 

A major impetus for MCS and other technological interventions is that the preservation and enhancement of life remains a priority in contemporary society [16]. However, it may be necessary to contain those desires within financial and scientific constraints of modern medicine, and to clearly delineate the goals of care in a given situation. With this, there is a greater onus on clinicians to help patients define what they hope to achieve by therapy such as MCS, how likely achieving that goal is, and what alternatives to MCS might be considered.

## 5. Future Directions: Promoting Autonomy through Shared Decision Making 

Patients with DT may experience improvement in physical, functional, and psychological well-being [20]. This comparison is often made to a patient's QOL before DT, which may be poor given the severity of advanced heart failure symptoms. When presenting patients with therapeutic options for advanced cardiac failure—a binary, black-and-white process is often perceived by patients. This includes the option to receive MCS and live, or the option to decline MCS and die [21]. Indeed, patients report feeling “cornered” into making decisions about MCS, as they see DT as “their only choice” versus the perception of the only alternative being certain death [22]. Attempts at normalizing a supportive and palliative care approach for patient with cardiovascular diseases has been helpful [23], and early addressing of potential complications of DT can be beneficial [24].

 Some patients or families fear trading one serious life-threatening illness for another after MCS implantation [22]. Though complication rates have improved with continuous-flow devices [3], bleeding, infection, end-organ (i.e., renal) failure and stroke can occur and have devastating consequences [3, 7, 25, 26]. As one group has stated, “cardiovascular care is increasingly complicated and requires striking balances between quality of life and longevity, high-tech interventions and supportive care” [27].

 Discussion regarding patient's values, preferences, and goals of care is critical to assuring a patient-centered outcome after DT. This is particularly important when complications occur or when surrogate decision-making is needed given suboptimal outcome. Studies have demonstrated that surrogates often underestimate patients' health care values, which can vary over time [28]. Furthermore, the benefits, burdens, and efficacy of therapies, particularly in the context of poor functional or cognitive outcomes vary highly among patients [29]. Patients with advanced heart failure may favor symptom control over survival [30], while others found 60% of patients with advanced heart failure expressed strongly polar opinions favoring treatments that promoted QOL or survival [31]. These factors are critical to framing how care preferences should be honored individually, but are important in promoting fiscal responsibility and resource stewardship at a macrolevel. A determination of “medical necessity” of DT is subject to an individual's bias, error or uncertainty [32]. Decision-making processes should be based on “evidence, reasons, and principles that all fair-minded people can agree are relevant” [33]. What defines medical necessity may vary in conceptualization and operationalization, but often circles back to greater core issues [32]: “what are the goals of medicine?” or “how important is cost containment” [9, 32]. 

 Although many patients experience improved QOL after MCS, DT may also prolong life without improving quality. As DT is an accepted standard of care available and indicated for use in the current US health care system, current medical decision-making efforts might be more effectively focus on promoting shared decision making in the context of presenting an accurate assessment of the risks, benefits, and alternatives to DT. However, clinicians may benefit from clearer guidelines regarding appropriate patient selection for MCS, including those most likely to benefit as well as those with psychosocial factors that might portend a poor outcome [34]. Cost-minimization strategies and cost-effectiveness analyzes may produce QALYs and cost-effectiveness data which guide macrolevel decisions or policies, but these fall short in assisting patients and clinicians with bedside decision making. Iterative deliberation regarding evolving best clinical practices and individual assessment of the medical appropriateness of MCS are needed to address these complex issues.

## 6. Conclusions

 The use of MCS to improve survival and QOL for patients with advanced heart failure continues to grow. Improvement in the technology, as well as increasingly favorable cost-effective analysis, suggests that DT may be a viable option for many patients with heart failure who are not transplant-eligible. This field is in a state of relative infancy, and we suspect much improvement in MCS technology and therapy will come forth with an improvement in overall costs. Studies continue to inform us about which patients are the best candidates for DT; nevertheless, determining an optimal MCS candidate is complex. We conclude that selection decisions regarding MCS may be best made on physiologic and psychosocial criteria, while emphasizing informed shared decision making. As these challenges continue to unfold, we believe ongoing dialogue and exploration of these ethical issues regarding justice will be critical in helping clinicians to determine best practices.

## Abbreviations

A4R: Accountability for reasonableness
DT: Destination therapy
INTERMACS: Interagency Registry for Mechanically Assisted Circulatory Support
MCS: Mechanical circulatory support
QOL: Quality of life
USD: United States dollars.
